# Influenza Gain-of-Function Experiments: Their Role in Vaccine Virus Recommendation and Pandemic Preparedness

**DOI:** 10.1128/mBio.02430-14

**Published:** 2014-12-12

**Authors:** S. Schultz-Cherry, R. J. Webby, R. G. Webster, A. Kelso, I. G. Barr, J. W. McCauley, R. S. Daniels, D. Wang, Y. Shu, E. Nobusawa, S. Itamura, M. Tashiro, Y. Harada, S. Watanabe, T. Odagiri, Z. Ye, G. Grohmann, R. Harvey, O. Engelhardt, D. Smith, K. Hamilton, F. Claes, G. Dauphin

**Affiliations:** ^a^ WHO Collaborating Center for Studies on the Ecology of Influenza in Animals, Department of Infectious Diseases, St. Jude Children’s Research Hospital, Memphis, Tennessee, USA; ^b^ WHO Collaborating Centre for Reference and Research on Influenza, Peter Doherty Institute for Infection and Immunity, Melbourne, Victoria, Australia; ^c^ WHO Collaborating Centre for Reference and Research on Influenza, Division of Virology, MRC National Institute for Medical Research, Mill Hill, London, United Kingdom; ^d^ WHO Collaborating Center for Reference and Research on Influenza, Chinese National Influenza Center, National Institute for Viral Disease Control and Prevention China CDC, Beijing, People’s Republic of China; ^e^ WHO Collaborating Centre for Reference and Research on Influenza, National Institute of Infectious Diseases, Laboratory of Influenza Virus Surveillance, Influenza Virus Research Center, Tokyo, Japan; ^f^ Division of Viral Products, Office of Vaccines Research and Review, Center for Biologics Evaluation and Research, Food and Drug Administration, Rockville, Maryland, USA; ^g^ Immunology and Vaccines, Therapeutic Goods Administration Laboratories, Woden, ACT, Australia; ^h^ National Institute for Biological Standards and Control, Medicines and Healthcare Products Regulatory Agency, Potters Bar, United Kingdom; ^i^ Center for Pathogen Evolution, Department of Zoology, WHO CC for Modeling Evolution and Control of Emerging Infectious Diseases, University of Cambridge, Cambridge, United Kingdom; ^j^ OIE Scientific and Technical Department, OIE, Paris, France; ^k^ OFFLU/EMPRES Laboratory Unit, Animal Health Service, FAO, Rome, Italy

## Abstract

In recent years, controversy has arisen regarding the risks and benefits of certain types of gain-of-function (GOF) studies involving avian influenza viruses. In this article, we provide specific examples of how different types of data, including information garnered from GOF studies, have helped to shape the influenza vaccine production process—from selection of candidate vaccine viruses (CVVs) to the manufacture and stockpiling of safe, high-yield prepandemic vaccines for the global community. The article is not written to support a specific pro- or anti-GOF stance but rather to inform the scientific community about factors involved in vaccine virus selection and the preparation of prepandemic influenza vaccines and the impact that some GOF information has had on this process.

## GUEST EDITORIAL

Influenza viruses are a major cause of morbidity and mortality worldwide. Each year, outbreaks of seasonal influenza viruses are estimated to result in 25 to 50 million cases in the United States alone, leading to over 200,000 hospitalizations and 30,000 to 40,000 deaths annually. Globally, the burden of disease is estimated to be as high as 1 billion cases annually (1), which can increase dramatically during a pandemic. Over the past decade, we have seen an increase in the detection and reporting of avian influenza (AI) viruses crossing the species barrier to infect humans, often resulting in severe and even fatal disease. Since the original reports of human infections with highly pathogenic avian influenza (HPAI) A (H5N1) viruses in 1997, a variety of both HPAI and low-pathogenicity avian influenza (LPAI) subtypes, including H5Nx, H7Nx, H9N2, H6N1, and H10N8 viruses, have caused human infections (2). To date, over 1,000 human AI cases have been reported to the World Health Organization (WHO), primarily associated with H5N1 or H7N9 viruses (3), with many of these infections being fatal. Recently, reports of humans coinfected with seasonal and avian influenza viruses have also occurred (4, 5). However, zoonotic influenza infections are not limited to avian viruses. Indeed, the first influenza pandemic of the 21st century was caused by zoonotic introduction of a virus, A (H1N1) pdm09, from swine (6, 7). In addition, an increasing number of human infections with variant H3N2 and H1Nx influenza viruses that circulated in swine populations have been reported since 2010 (2), most of which were in children in contact with pigs at agricultural fairs in the United States (8). These types of infections in an immunologically naive human host raise public health concerns about the emergence of a zoonotic virus with the ability to effectively transmit between humans, resulting in the next pandemic.

The primary public health medical countermeasure against influenza is vaccination. Although the first inactivated influenza vaccines were introduced in the 1940s, it soon became evident that influenza viruses undergo antigenic changes that reduce vaccine efficacy. Surveillance networks were soon developed to identify the emergence of antigenically drifted viruses and identify a representative candidate vaccine virus (CVV) for use in vaccine manufacturing. Since 1952, the WHO has coordinated monitoring of the antigenic properties of influenza viruses in humans and, more recently, in animals within the Global Influenza Surveillance Network (GISN), which in May 2011 was renamed the Global Influenza Surveillance and Response System (GISRS). The GISRS is currently comprised of 6 WHO collaborating centers (CCs) (located in Melbourne, Australia, Beijing, China, Tokyo Japan, London, United Kingdom, the Centers for Disease Control and Prevention, Atlanta, GA, USA, and St. Jude Children’s Research Center, Memphis, TN, USA), 4 essential regulatory laboratories (ERLs), 141 national influenza centers (NIC) in 111 WHO member states, and 13 H5 reference laboratories. GISRS also collaborates closely with the animal health sector, including the Food and Agriculture Organization (FAO), the World Organization for Animal Health (OIE), and the OFFLU (the joint OIE-FAO network of animal influenza experts). Throughout the year, samples are collected from patients with influenza-like illness or acute respiratory infection and sent for assessment of influenza infection and virus isolation. Isolated viruses undergo detailed characterization, including genetic, antigenic, antiviral drug susceptibility, and molecular analyses. Influenza surveillance is also conducted in the animal sector, including in healthy animal populations. Twice yearly, the WHO GISRS subject matter experts meet to review data in the context of CVV selection. Seasonal influenza viruses in circulation in the months leading up to September are reviewed to identify viruses with distinct antigenic characteristics and associated epidemiological features that would justify a change in the current recommended CVVs for use in the following year in the Southern Hemisphere, and those in circulation in the months leading up to February are reviewed for the recommendation of viruses to be included in vaccines for the following winter in the Northern Hemisphere. At the same meetings, an expanded group including animal health experts also reviews comprehensive data about zoonotic influenza infections, outbreaks of influenza in animals, and virologic characteristics of corresponding influenza viruses to determine if one or more CVVs should be developed for pandemic preparedness. It should be noted that CVVs for zoonotic viruses are recommended primarily if these subclades have been associated with human infections. For seasonal influenza vaccines, from the time these recommendations are made, manufacturers require 6 months to produce vaccines and begin distribution. An overview of the annual production cycle for influenza vaccine can be found in reference 9. For viruses with pandemic potential, CVVs are made available to manufacturers for pilot studies related to vaccine production (10), and in some instances, such as for the A (H7N9) outbreak in China, clinical trial lots are produced and vaccine stockpiles made (11; http://www.hhs.gov/nvpo/nvac/meetings/pastmeetings/2014/barda-vaccines-february2014.pdf).

The remainder of this article will focus on how information garnered from gain-of-function (GOF) studies helps to inform the influenza vaccine strategy for pandemic preparedness, from selection of candidate vaccine viruses and development of high-yield seeds to manufacture of safe vaccines for the global community; the needed information includes mutations identified by virulence and transmission studies with A/H5N1 viruses, identification of important antigenic sites through vaccine escape studies, basic research on the hemagglutinin (HA) region associated with virulence in poultry, and ways to enhance growth in culture systems.

## INFORMING THE DECISION PROCESS: THE INFLUENZA A/H5 VIRUSES

To understand how information from GOF studies has aided the decision-making process, we will focus on data from zoonotic viruses, specifically HPAI H5N1 viruses. Since emerging in 1997, the HPAI H5N1 viruses have spread geographically and become endemic in poultry in several parts of the world where zoonotic infections continue to occur. As of today, the HA genes of these viruses have evolved from the original A/goose/Guangdong/1/96 lineage, resulting in 10 unique first-order HA clades, with most containing numerous subclades comprised of genetically and antigenically distinct viruses (12). To keep up with the rapid antigenic variation associated with the genetic divergence of H5N1 viruses identified in recent years, 26 CVVs have been generated by members of the GISRS (13). While some of these CVVs have been developed and placed in institutional libraries for future use, several have been used to produce small-scale (pilot) lots and/or have been produced as manufacturer-scale lots for clinical trial evaluation and long-term storage as bulk antigen for formulation into vaccine if required, as part of government-funded stockpiles (14). Selection of viruses chosen for CVV generation, pilot lot production, and, ultimately, inclusion in the national or global stockpiles is often made with consideration of GOF mutations identified during molecular risk assessment so that viruses with the greatest pandemic potential are selected. How is this accomplished?

**FIG 1:**
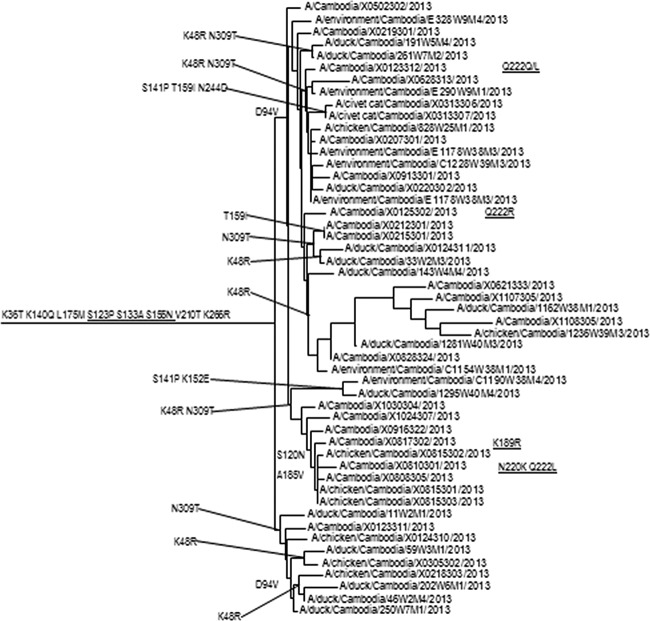
Phylogenetic analysis of Cambodian HPAI H5N1 clade 1 viruses. Phylogenetic tree of Cambodian HPAI H5N1 clade 1 HA genes as rooted to the A/Vietnam/1203/2004 CVV.

At the twice-yearly WHO Consultation on the Composition of Influenza Vaccines meeting, genetic and antigenic information from H5Nx viruses (and other zoonotic subtypes) isolated from infected animals and humans is analyzed to determine if new CVVs are required. Specifically, the HA sequences of related viruses are aligned and compared to available CVVs (Fig. 1) to determine genetic heterogeneity. Antigenic variation is assessed with hemagglutination inhibition assays (HIA) that measure the ability of postinfection ferret antisera, raised against panels of CVVs and related wild-type viruses, to inhibit the agglutination of red blood cells by the field isolates (Table 1). An 8-fold or greater reduction in HIA titers compared to the titers observed with the homologous virus is considered to indicate a significant change in antigenicity, potentially warranting generation of a new CVV. Given the continual evolution of the H5N1 viruses, the appearance of numerous zoonotic infections, and the laboratory resources required to produce a CVV, risk assessment considerations are critically important for proposing the generation of a new CVV. To support this decision making, amino acid differences in the mature HA1 proteins of previously detected viruses, circulating viruses, and existing CVVs are compared to identify molecular correlates of antigenic variation detected by HIA. Additionally to the identification of amino acid substitutions in antigenic sites, identification of substitutions previously shown to cause a GOF phenotype, such as changes associated with increased binding to mammalian receptors and transmission between mammals (reviewed in references [Bibr B15][Bibr B16][Bibr B18]), is undertaken to weigh their potential public health significance or risk (Table 2). While the ability to link influenza virus genotype to phenotype is suboptimal and much more data are required, attention to mutations specifically identified by GOF studies allows experts to assess the relevance of specific molecular determinants in relation to virologic and epidemiological factors considered for pandemic preparedness and is of particular relevance for decisions relating to the production of manufacturing seeds and trial lots and the stockpiling of vaccines or antigen.

**TABLE 1:** Hemagglutination inhibition assay of circulating strains of H5N1 viruses collected from humans in Cambodia^*a*^

Antigen(s)	Clade	HIA titer of ferret antiserum^*b*^						
		VN/1203	CB/R0405050	CB/W0526301	CB/W0329318	CB/X0123311	CB/X0810301	CB/X0810301 RG34B
Reference antigens								
A/Vietnam/1203/2004	1	160	40	320	80	80	80	40
A/Cambodia/R0405050/2007	1	40	160	20	20	20	10	20
A/Cambodia/W0526301/2012	1.1.2	80	40	640	80	320	160	160
A/Cambodia/W0329318/2012	1.1.2	160	40	160	160	80	40	40
A/Cambodia/X0123311/2013	1.1.2	80	20	320	80	320	160	160
A/Cambodia/X0810301/2013	1.1.2	40	40	320	80	160	160	160
A/Cambodia/X0810301/2013, A/Cambodia/X0810301/2013, PR8-IDCDC-RG34B	1.1.2	160	160	1,280	160	640	320	320
Test antigens								
A/Cambodia/W0112303/2012	1.1.2	80	20	80	40	40	20	10
A/Cambodia/X0817302/2013	1.1.2	20	80	160	20	320	80	80
A/Cambodia/X0628313/2013	1.1.2	80	40	320	80	320	320	160
A/Cambodia/X0828324/2013	1.1.2	80	40	320	80	320	160	160
A/Cambodia/X0125302/2013	1.1.2	160	80	640	80	640	160	320
A/Cambodia/X0916322/2013	1.1.2	80	40	640	80	640	160	320

^*a*^ Postinfection ferret antisera were raised against the reference viruses indicated. For A/Cambodia/X0810301/2013, antisera were raised against the wild-type virus and the CVV based on a PR8 backbone; both antisera showed good, and comparable, reactivities with the majority of test viruses, indicating the suitability of the CVV for vaccine manufacture. Underlined values indicate titer against homologous virus.

^*b*^ Names of viruses are abbreviated and truncated. CB, Cambodia; VN, Vietnam.

**TABLE 2:** Amino acid differences between circulating strains of H5N1 viruses collected from humans in Cambodia and candidate vaccine viruses

Position in mature H5 HA1	Amino acid(s) in:											Annotation(s)^*a*^
	A/Vietnam/1203/2004	A/Cambodia/R0405050/2007	A/Cambodia/V0417301/2011	A/Cambodia/W0526301/2012	A/Cambodia/W0329318/2012	A/Cambodia/X0123311/2013	A/Cambodia/X0810301/2013	A/Cambodia/X0817302/2013	A/Cambodia/X0621333/2013	A/Cambodia/X0125302/2013	A/Cambodia/X0123312/2013	
14	E		K		K							
36	K	T	T	T	T	T	T	T	T	T	T	
48	K		R	R	R	R	R	R	R	R	R	Antigenic site E
86	V				I							
94	D		V	V	V	V	V	V	V	V	V	
120	S						N	N				
123	S	P	P	P	P	P	P	P	P	P	P	Antigenic site B; increased virus binding to α2-6
133	S	A	A	A	A	A	A	A	A	A	A	Antigenic site A; receptor binding; increased pseudovirus binding to α2-6
140	K			Q		Q	Q	Q	Q	Q	Q	Antigenic site A
155	S	N				N	N	N	N	N	N	Antigenic site B; increased virus binding to α2-6
175	L	M	M	M	M	M	M	M	M	M	M	
185	A						V	V				Antigenic site B
186	E										A	Antigenic site B
189	K	N						R				Antigenic site B; increased virus binding to α2-6
210	V	T	T	T	T	T	T	T	T	T	T	Antigenic site D
213	I								T			
220	N						K					Receptor binding; N220K with N154D, Q222L, and T315I to H5 HA virus transmissible among ferrets
222	Q						L			R	Q/L	Receptor binding; antigenic site D; increased virus binding to α2-6
266	K			R	R	R	R	R	R	R	R	
309	N		T	T	T	T	T	T	T	T	T	

^a^ Underlined annotations indicate substitutions identified by GOF studies and included in the H5N1 genetic change inventory.

In a recent example, HA gene mutations were detected in clade 1 H5N1 viruses causing human infections in Cambodia that were associated with alteration of receptor-binding specificity and increased respiratory droplet transmission in ferrets (19). Amino acid sequence comparisons of viruses isolated in 2013 to clade 1 progenitor strains revealed that all viruses shared amino acid substitutions at four positions (HA S123P, S133A, S155N, and K266R) (Table 2). Three of these four HA substitutions were shown in GOF experiments to increase binding of H5N1 viruses to mammalian host cell α2,6-linked sialic acid receptors either alone (S133A, S155N) or in combination with other mutations (S123P) (20–22). In addition, several individual viruses from human cases had other HA amino acid substitutions experimentally linked to increased binding to α2,6-linked sialic acid receptors (K189R, Q222L) and enhanced respiratory droplet transmission of a clade 1 virus in a ferret model (N220K with Q222L) (18, 20, 23). The detection of these amino acid signatures coincided with the abrupt rise in human cases in Cambodia from 11 between 2005 and 2012 to 26 in 2013, although no causality was determined (24). Nevertheless, these findings suggested that a new CVV would be useful for pandemic-preparedness purposes, and the WHO expert group decided to develop a CVV from A/Cambodia/X0810301/2013. This virus possessed two of the markers described by Imai et al. (18) as enhancing aerosol transmission of a clade 1 virus between ferrets, as well as three substitutions shown to increase binding to the α2,6-linked sialic acid receptor. While the biological significance of the Imai et al. mutations may be dependent on the specific genetic backbone of the virus tested for GOF (i.e., clade 1 A/Vietnam/1203/2004), the discovery of these mutations in a related clade 1 virus was of significant concern. Subsequent antigenic analysis with ferret antisera raised against the wild type and CVV showed the CVV to be antigenically equivalent to other H5N1 viruses circulating in Cambodia and Vietnam (13) at that time and that it would therefore serve as an appropriate vaccine for pandemic preparedness purposes (Table 1). Although a CVV against clade 1.1.2 viruses, of which A/Cambodia/X0810301/2013 is an example, was proposed at that time due to the increased human cases and observed antigenic variation between the 2013 viruses and previous CVVs, it was determined that this particular CVV should be developed with a priority over others belonging to different clades. Without GOF data, there would not have been a reason to design a CVV against this specific parental strain.

## FROM LETHAL POULTRY VIRUS TO LIFE-SAVING VACCINES

The great majority of influenza vaccines are prepared from viruses propagated in embryonated chicken eggs, which are then inactivated with either formaldehyde or β-propiolactone and either split by detergent or subjected to surface antigen purification in accordance with WHO recommendations. Producing vaccines from egg-propagated wild-type HPAI viruses is problematic because the rapid killing of chicken embryos leads to low vaccine yields, poor-quality harvests, and biosafety issues. Work with HPAI viruses requires enhanced containment facilities, which very few vaccine manufacturers possess, and moreover, production staff would be immunologically naive to such viruses (25, 26). Consequently, viruses must be genetically modified to allow the safe production of prepandemic vaccines targeting HPAI.

**FIG 2:**
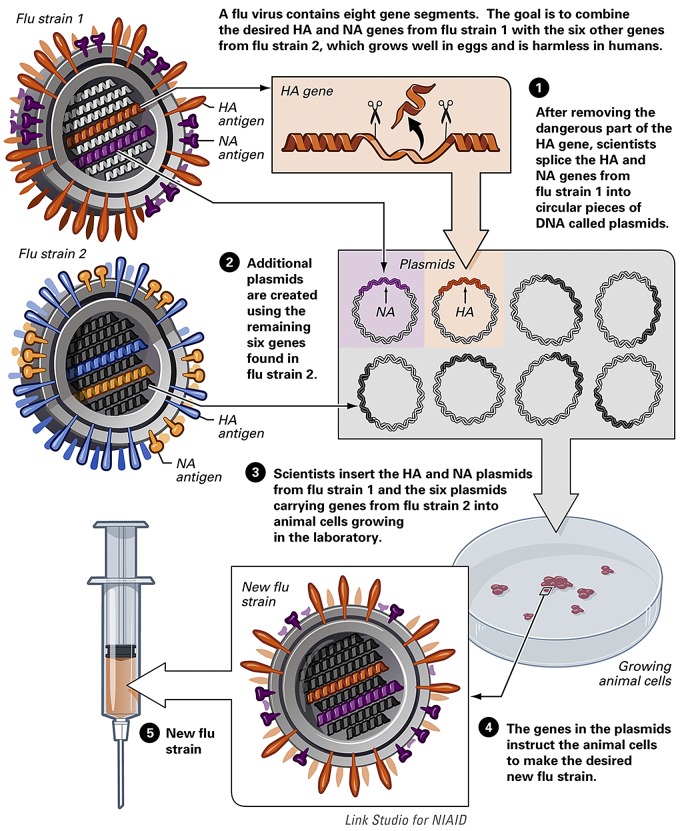
Generation of recombinant vaccines for influenza by reverse genetics. From the National Institute of Allergy and Infectious Diseases.

Currently, HPAI and many other zoonotic CVVs are produced using reverse genetics (RG) based on the WHO-approved A/Puerto Rico/8/34 (PR8) virus. PR8 was isolated from a human and has been propagated extensively in eggs and other laboratory systems to yield a virus that is highly attenuated for humans but grows to high titers in eggs (25). We know from studies of viral pathogenicity beginning in the 1970s that the major virulence determinant in HPAI H5 viruses is a short span of basic amino acids at the HA1/2 cleavage site which allows the HA protein to be easily cleaved (activated) by host cell proteases that are widely distributed through the body (reviewed in reference 27). Using genetic engineering techniques, the nucleotides encoding the extra basic amino acids at the cleavage site are removed from the HA gene segment. To generate a candidate vaccine virus that can be used safely by manufacturers, the modified HA gene and the NA gene from the HPAI H5 CVV are expressed on a PR8 backbone by RG (Fig. 2). This is a process whereby vaccine viruses can be produced “synthetically” using a series of bacterial plasmids which are introduced into mammalian cells to produce viable, infectious, influenza virus (28). The 6:2 RG virus (6 PR8 genes and 2 from the HPAI H5 virus of interest) is then produced under high biosafety containment conditions, and safety is tested by assessing viral growth in the absence of trypsin, the ability of the virus to cause chicken embryo death, intravenous chicken pathogenicity, and attenuation in ferrets (29). If the RG virus passes all of these tests, the virus is no longer considered HPAI and can be distributed as a CVV for safe use in manufacturing, which is performed under biological containment. To date, this process has been used to provide 26 pandemic H5 CVVs that are available for pilot lot vaccine production, with 4 more CVVs in preparation (13). This example illustrates how knowledge derived from research into basic markers of viral pathogenicity identified the multibasic cleavage site of HA as a pathogenic determinant that, subsequently, upon generation of more-advanced technologies, led to innovative vaccine design with long-term benefits to public health.

## EDUCATING THE DECISION PROCESS: SEASONAL VIRUSES AND ANTIGENIC DRIFT

The ability of the HA protein to evade antibodies through “antigenic drift” drives the need to update seasonal influenza vaccines. Antigenic drift is caused by amino acid substitutions in HA epitopes recognized by antibodies that neutralize viral infectivity by blocking HA interaction with host cell sialic acid receptors. Much of our understanding of the HA alterations associated with evasion of host antibody responses has come from characterizing vaccine escape mutant viruses generated in the laboratory, a precursor of GOF work that began in earnest during the 1970s along with genetic sequencing. These studies have provided invaluable information on the importance of the antigenic sites on HA recognized by neutralizing antibodies, specific amino acid alterations associated with loss of antigenicity, and the importance of glycosylation, and they have demonstrated that viruses with such changes frequently circulate in nature (30–36). During the WHO discussions on influenza viruses circulating in humans (influenza A/H1N1, A/H3N2, and influenza B viruses), the antigenic properties of several thousand circulating viruses are compared with those of previously circulating viruses and current vaccine viruses, which is similar to the process described above for the H5 viruses. To support these antigenic data, the HA and neuraminidase (NA) gene sequences are determined for many hundreds of the recently circulating viruses to allow comparisons to be made between the amino acid sequences of the HA and NA proteins of circulating viruses and those of potential vaccine viruses. Any amino acid differences known to be associated with altered antigenic function(s) are taken into consideration to inform the decision process. In summary, a large body of knowledge gained through GOF studies of antigenic traits has informed vaccine development for seasonal and pandemic influenza.

## HGR VIRUSES: WITHOUT THEM THERE MAY NOT BE ENOUGH INFLUENZA VACCINE

Among the most important GOF work for the vaccine production process is the generation of high-growth or high-yield reassortant (HGR or HYR) CVVs (37). Generally, human influenza virus isolates grow poorly in embryonated chicken eggs. In contrast, PR8 not only grows to very high titers in eggs, it also confers the ability for improved growth in eggs to other viruses if genes of the wild-type virus are replaced with the corresponding PR8-derived genes. Consequently, gene reassortment of wild-type influenza A viruses is performed with the PR8 virus to provide HGR/HYR CVVs for seasonal vaccine production. These HGR/HYR viruses must contain the HA and NA genes of the wild-type virus and one to six of the PR8 internal genes. Without these HGR/HYR viruses, which could be considered GOF viruses by the strictest definition, vaccine manufacturers would be unable to provide sufficient vaccine to meet demand each year. Such a shortage occurred in 2004, which led to ethical discussions on ways to prioritize who should receive the available vaccines (38).

In summary, information garnered from basic influenza virus research, including GOF studies, focused on specific viral traits, such as receptor binding, transmissibility, pathogenicity, antigenicity, and antigen yield in eggs, has benefited public health and the vaccine production process in numerous ways. These studies help to define molecular markers associated with mammalian infection, evasion of neutralizing antibodies, and attenuation and/or replication in various hosts, thereby informing vaccine recommendations to aid in vaccine design and production. Frequently, this knowledge has been derived from basic research studies that relied on GOF to test a hypothesis; however, the insights that ultimately benefitted public health often were not appreciated until much later. With the continual evolution of influenza viruses and the potential for zoonotic events leading to pandemics, we must remain vigilant. For this reason, care must be taken in considering the regulation of GOF studies of influenza viruses to avoid unforeseen consequences for global public health and to ensure the safe and ongoing provision of essential vaccines and public health countermeasures.
